# Assessment of XCI skewing and demonstration of XCI escape region based on single-cell RNA sequencing: comparison between female Grave’s disease and control

**DOI:** 10.1186/s12860-025-00533-z

**Published:** 2025-01-31

**Authors:** In-Cheol Baek, Soo Yeun Sim, Byung-Kyu Suh, Tai-Gyu Kim, Won Kyoung Cho

**Affiliations:** 1 Catholic Hematopoietic Stem Cell Bank, College of Medicine, The Catholic University of Korea, Seoul, Republic of Korea; 2 Department of Pediatrics, Seoul St. Mary’s Hospital, College of Medicine, The Catholic University of Korea, Seoul, Republic of Korea; 3 Department of Microbiology, College of Medicine, The Catholic University of Korea, Seoul, Republic of Korea; 4 Department of Pediatrics, College of Medicine, St. Vincent’s Hospital, The Catholic University of Korea, 93, Jungbu-daero, Paldal-gu, Suwon-si, Seoul, Gyeonggi-do 16247 Republic of Korea

**Keywords:** X chromosome inactivation, Autoimmune thyroid disease, Single nucleotide polymorphism, Single-cell RNA sequencing, XCI skewing and escape

## Abstract

**Background:**

The reactivation and loss of mosaicism hypothesis due to X chromosome inactivation (XCI) skewing and escape could influence gender differences in autoimmune diseases. XCI selectively inactivates one of the two X chromosomes in females.

**Methods:**

To estimate XCI skewing and the occurrence of XCI escape, we conducted a normal female (NF) without a history of autoimmune thyroid disease (AITD) and a patient with Grave’s disease (GD) based on a thyroid diagnosis. After single-cell RNA sequencing, heterozygous variants were converted and transformed. XCI skewing was calculated using the formula and the skewing degree was defined. NF/GD genes were compared using correction methods. Positions are heterozygous within a single cell as indicated by a unique barcode.

**Results:**

XCI skewing showed 45.8%/48.9% relatively random, 29.4%/27.0% skewing, 24.6%/23.7% severe skewing, and 0.2%/0.4% extreme severe skewing. 24.8%/24.1% in NF/GD exhibited severe skewing or higher. A total of 13 genes were significantly associated with XCI skewing ratios in NF/GD cells. In total, 371/250 nucleotide positions with only one barcode (representing a unique cell) were identified for XCI escape. A total of 143/52 nucleotide positions spanned 20/6 genes, and 12/1 genes were identified as XCI escapes.

**Conclusions:**

These results could aid in understanding the immunogenetics of gender differences in various autoimmune disease pathophysiologies.

**Supplementary Information:**

The online version contains supplementary material available at 10.1186/s12860-025-00533-z.

## Introduction

The X chromosome contains about 1,000 genes—vastly more than the Y chromosome—including immune response genes and proteins, receptors, and regulators [1]. One of the two copies in the X chromosome is inactivated to balance the allele capacities between females and males [2, 3]. The X chromosome inactivation (XCI) process is regulated by the X inactive specific transcript (*XIST*) gene, which is transcribed and expressed only by the inactivated X chromosome [4, 5]. However, the genes that escape XCI are 15 to 20% in the X chromosome [6] In addition, a phenomenon known as XCI skewing generally indicates preferentially specific XCI with maternal or paternal origin XCI.

The dominance of autoimmune diseases in women has long been recognized, with the most marked gender differences observed in Grave’s disease (GD) [7, 8]. Recently, much attention has been paid to direct genetic differences such as sex-specific regulation of sex hormones and immune-related genes as well as of X chromosomes and related genes [2]. The differences are essential for developing personalized treatment strategies and improving patient outcomes in autoimmune diseases [9]. They often exhibit significance in their pathogenesis, progression, and treatment outcomes such as Sjögren’s syndrome (SS), systemic lupus erythematosus (SLE), rheumatoid arthritis (RA), and multiple sclerosis (MS) [10–14]. The exact cause is believed to result from a mix of genetic, hormonal, and environmental factors [7, 8]. The reason polymorphic genes exist on the X chromosome is that the allele and genotype frequencies between women and men are different, which can lead to distinct phenotypic effects and the female advantage in the host response to injury and infection [15]. In addition, inactivation and skewing of the X chromosome can lead to an increased risk of RA and autoimmune diseases in women [16–18]. To assess XCI escape and skewing, it is challenging to differentiate using bulk sequencing. Therefore, single-cell RNA sequencing (scRNA-seq) is essential for accurate identification and analysis [4, 19–21]. The droplet-based system can count tens of thousands of single cells per sample for 30 mRNA [19]. Research groups have systematically used scRNA-seq and the 10x Genomics platform to estimate the allele-specific expression of heterozygous variants [4, 20]. The systematic investigation of XCI incorporates transcripts from individuals, encompassing GTEx tissues [21].

The use of T-cells and B-cells has been proposed to explain the increase in female immune response. CD4 (+) T-cells, which are important components of the adaptive immune response, consist of approximately three-quarters of all lymphocytes. They are known to play key roles in pathogen clearance, autoimmune disease control, and pathogenic cell clearance [22].

Previous studies have primarily used bulk sequencing methods to explore XCI skewing and escape. These approaches have provided valuable insights, but may be associated with certain limitations. In this study, we investigate the degree of XCI skewing and identify XCI escape using scRNA-seq analysis. Our results aim to demonstrate how the current approach addresses some of the challenges posed by existing methods.

## Materials and methods

### Subjects

In November 2022, a healthy Korean female volunteer without autoimmune thyroid disease (AITD) was selected as the normal female (NF) control. The NF volunteer underwent a medical interview and examination to confirm the absence of significant health issues. One GD patient was included, diagnosed based on thyroid imaging, iodine uptake, hyperthyroid symptoms, and TSH receptor antibodies. Patients with other autoimmune, hematologic, or endocrine diseases, or insufficient blood samples, were excluded. All participants provided informed consent for this genetic study. This study was approved by the Institutional Review Board (IRB) of The Catholic University of Korea (IRB Number: VC17TESI0129), Seoul, Korea, and the study were conducted in accordance with the Declaration of Helsinki. Peripheral blood mononuclear cell (PBMC) samples were obtained from NF and one patient diagnosed with GD (Table 1) for use in scRNA-seq. PBMCs were purified using Ficoll-Hypaque (GE Healthcare, Pittsburgh, PA, USA). To prevent nonspecific binding, 2.5 µL of Fc Block (BD Pharmingen™; 564220) per 1 × 10⁵ cells was added to each prepared sample and incubated at room temperature for 10 min. Following this, the cells were stained with fluorochrome-conjugated antibodies targeting CD3 (BioLegend; 300434), CD4 (BioLegend; 300514), CD14 (BioLegend; 325604), and CD45 (BioLegend; 304014) in the dark for 30 min. Flow cytometric analysis was subsequently performed using the FACS Canto system (BD Biosciences, San Jose, CA, USA) (Fig. S1). CD4+ T cells were isolated using magnetic microbeads (AutoMacs Pro separator; Miltenyi Biotec GmbH, Bergisch Gladbach, Germany), and the purity of the CD4+ T cells was confirmed via flow cytometric analysis [23].

**Table 1:** Characteristics of NF and GD

	NF	GD
Sex	F	F
Age at enrollment (years)	43	17
Age at diagnosis (years)	N/A	16
Goiter	Negative	Positive
T3 at diagnosis, 0.78–1.82 ng/mL	WNL	1.85
Free T4 at diagnosis, 0.85–1.86 ng/dL	WNL	2.03
TSH at diagnosis, 0.17–4.05 mIU/L	WNL	0.003
TSHR Ab positive at diagnosis	Negative	Positive
Clinically evident TAO (NOSPECS class II or higher)	Negative	Positive

*Abbreviations NF* normal female, *GD* Graves’ disease, *TSH* thyroid stimulating hormone, *TSHR Ab* TSH receptor antibody, *TAO* thyroid associated ophthalmopathy, *WNL* with in normal range

### Single-cell library preparation and scRNA-seq

A Chromium Controller (10× Genomics, San Francisco, CA) was used to performed scRNA-seq. The Chromium Single Cell Gene Expression Solution with Chromium Single Cell 3′ GEM, Library and Gel Bead Kit v2 (10× Genomics) was used for library preparation and processing following the manufacturer’s specifications [24]. Briefly, a single gel bead containing a single cell, reagent, and barcode oligonucleotide was encapsulated into a nanoliter-scale gel bead in emulsion (GEM). One thousand of the isolated CD4 T + cells were suspended in master mix solution. The cells were loaded into a well of the channel of a single-cell A chip. Gel beads and partitioning oil were added to another channel well. Single-cell beads in an emulsion mixture were produced by a Chromium Controller. We sorted CD4+ T cells from PBMCs and confirmed their viability. Then, the sorted cells were placed in RPMI media and transferred to Macrogen Inc. The composition of RPMI media is RPMI media (Gibco, Grand Island, NY, USA) supplemented with 10% fetal bovine serum (FBS; Gibco), 1% L-glutamine (Lonza, Walkersville, MD, USA), and 1% penicillin–streptomycin (Lonza). The cells were QC-processed with acridine orange / propidium iodide (AO/PI) and trypan blue (TB). AO/PI assessed the cell status and viability with the Luna-FL™ automated cell counter (Logos Biosystems, Gyeonggi-do, South Korea). In our experiment, we did not isolate RNA, but loaded the sorted cells onto the chromium device to form GEMs and synthesize cDNA. Then, scRNA-seq was performed according to the manufacturer’s instructions. After the single cells were lysed, reverse transcription and RNA barcoding were performed using oligo dT with unique molecular identifiers (UMIs) in emulsion. By dividing thousands of cells into nanoliter-scale GEM, all of the produced DNA molecules shared a 10-fold barcode. Each cell and transcript were uniquely barcoded with a UMI. The transcripts were amplified to the barcoded cDNA by 12 cycles of the thermocycler machine (Macrogen Inc., Seoul, Korea). The cDNA molecules were pooled and then ligated by an adapter. The products were purified and concentrated via PCR to create a final cDNA library. Purified libraries were quantified using qPCR according to the qPCR Quantification Protocol Guide (KAPA) and were qualified using TapeStation 4200 (Agilent Technologies, Inc, Santa Clara, CA). The libraries were sequenced using the Illumina HiSeq 4000 platform (Macrogen Inc.) according to the read length. Sequencing was performed using paired-end reads from Read 1 and Read 2 at the ends of the fragments [25, 26].

### Data collection for genes from the X chromosome

The Cell Ranger Single Cell v2.1.1 software (10X Genomics), an analysis pipeline, was used to process the sequenced data, barcodes, UMI, and gene counting and mapping [27]. Using Cell Ranger’s mkfastq, raw BCL files were demultiplexed into FASTQ files [28]. The FASTQ files were transferred to BAM (Binary Alignment Map) format. In Ubuntu-based Linux PC, all RNA sequencing data were obtained in “possorted_genome_bam.bam” and “possorted_genome_bam.bai” formats. The BAM files were sorted to “chrX_only.bam” and were indexed and phased by the program: “samtools (Tools for alignments in the SAM format) version: 1.13”. The phased files were transferred to text files named “phased_output.txt” (Table S1). The data processing codes of the number of barcode and barcoding frequencies were run to distinguish heterozygous and homozygous alleles of SNPs in the text files (Table S2). A summary of all sequencing parameters was evaluated by the Cell Ranger software (10x Genomics). Differential gene expression between cell groups was analyzed using the negative bi-nomial exact test (sSeq method) implemented in the Cell Ranger program. Cell Ranger compares the identified cell cluster to each other to determine genes highly expressed in a cluster with respect to other clusters. In NF/GD, a total of 1,031/1,909 cells were processed in Og-NSCs (3D_cellRanger), with 450,418/211,668 median reads per cell and 2,906/2,628 median genes per cell.

### Calculation of XCI skewing and escape

XCI skewing was calculated using the following formula MAX (allele 1, allele 2)/(allele 1 + allele 2) × 100 in MS Excel. We defined skewing as follows: 50–70% relatively random, 70–80% skewing, 80–90% severe skewing, and 90–100% extreme severe skewing [18, 29–33]. In addition, we considered positions that were heterozygous within a single cell, indicated by a unique barcode, as XCI escape. If the number of barcodes was just one in the heterozygous alleles with a barcoding frequency greater than or equal to 10 and less than or equal to 1,000 and the count of valid reads was greater than or equal to 3, we defined it as XCI escape, one of which was X1 and the other X2.

### Statistical analysis

Statistical significance was assessed using the Z-score test and the χ^2^ test. *P* values were corrected by the Bonferroni, Hochberg, Hommel, and false discovery methods, and odds ratio (OR) was calculated using Haldane’s modification of Woolf’s method [34–50]. The OR was used to explain whether cell-to-cell phenomena are likely to be involved in disease and association. A two-tailed *p*-value of < 0.05 was considered statistically significant.

## Results

### Data interpretation for assessment of XCI skewing

**Fig. 1:**
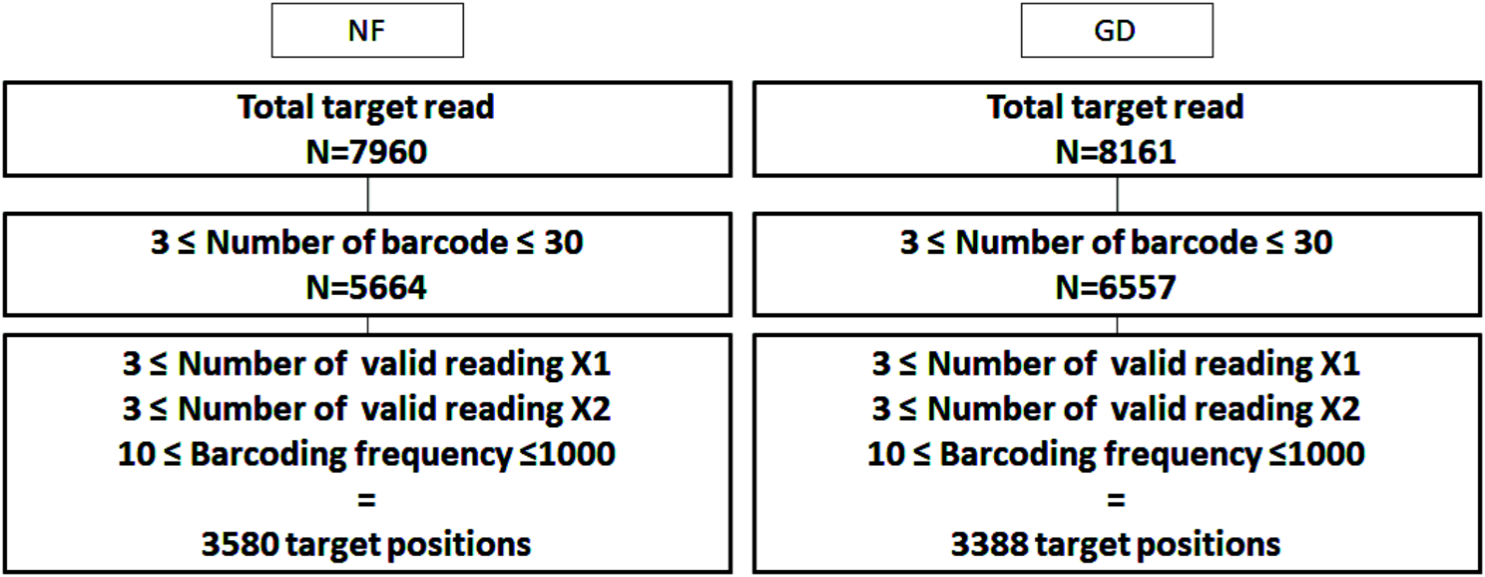
Strategy collected from total target read to target positions of NF and GD for XCI skewing. A standardized strategy for assessing XCI skewing was developed. The analysis considered barcoding frequencies ranging from 10 to 1,000, barcode counts of 3 to 30, and at least three effective supporting reads from both alleles. The filtered counts for single-cell gene expression analysis showed an increased the number of barcodes in GD, but when analyzing the target location, the number was similar to NF: 7,960/8,161 for total target reads, 5,664/6,557 for the number of barcodes, and 3,580/3,388 for target positions. NF, normal female; GD, Grave’s disease; X1, one allele of heterozygous genotype; X2, another allele of heterozygous genotype

For interpretation of data to assess XCI skewing and to reveal the XCI escape region, we used FASTQ files converted into BAM format and subsequently transformed into Samtool-phase files with phasing of heterozygous variants. We obtained 7,960/8,161 target nucleotide positions in NF/GD cells, respectively. After next generation sequencing, we prepared a strategy for analyzing the NF/GD data. Common criteria were established for analyzing XCI skewing. We analyzed the criteria with a barcoding frequency from 10 to 1,000 times, barcode counts between 3 and 30, and more than three effective supporting reads from both alleles, focusing on 3,580/3,388 positions (Fig. 1). For NF/GD, one allele of the heterozygous genotype (X1) was 3,580/3,380 of target positions, 3/3 of minimum, 80/83 of maximum, 11.80/9.64 of mean, and 10.97/8.97 of standard deviation. Another allele of the heterozygous genotype (X2) was 3,580/3,380 of target positions, 3/3 of minimum, 110/91 of maximum, 11.53/9.91 of mean, and 11.59/9.46 of standard deviation. Percentage (%) was 3,580/3,380 of target positions, 50/50 of minimum, 94.7/92.9 of maximum, 70.00/69.33 of mean, and 10.85/11.17 of standard deviation. Barcoding frequency was 3,580/3,380 of target positions, 10/10 of minimum, 322/199 of maximum, 42.23/34.02 of mean, and 37.03/26.56 of standard deviation. The barcoding number was 3,580/3,380 for target positions, 3/3 for minimum, 30/30 for maximum, 7.87/9.20 for mean, and 5.50/5.93 for standard deviation (Table 2).

**Table 2:** Data interpretation for assessment of XCI skewing

	NF/GD				
	Target positions	Minimum	Maximum	Mean	Standard deviation
X1	3,580/3,380	3/3	80/83	11.80/9.64	10.97/8.97
X2	3,580/3,380	3/3	110/91	11.53/9.91	11.59/9.46
%	3,580/3,380	50/50	94.7/92.9	70.00/69.33	10.85/11.17
Barcoding frequency	3,580/3,380	10/10	322/199	42.23/34.02	37.03/26.56
Number of barcode	3,580/3,380	3/3	30/30	7.87/9.20	5.50/5.93
Validation	3,580/3,380				

NF, normal female; GD, Grave’s disease; X1, one allele of heterozygous genotype; X2, another allele of heterozygous genotype

**Fig. 2:**
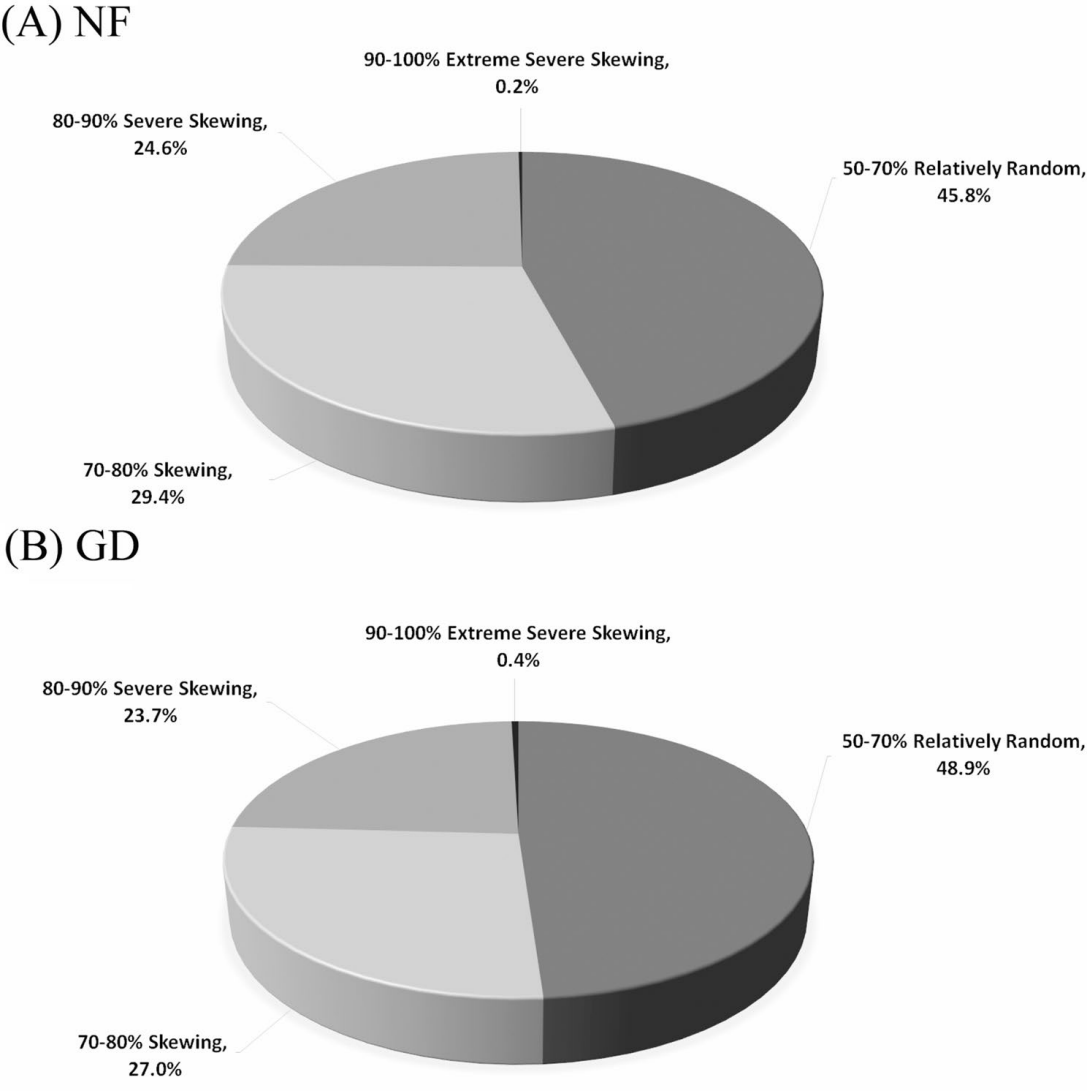
Distribution of XCI skewing degrees in NF and GD cells, based on 3,580/3,380 target nucleotide positions. We defined the degree of XCI skewing. They were categorized as follows: Relatively Random (50–70%): 45.8% (NF) / 48.9% (GD), Skewing (70–80%): 29.4% (NF) / 27.0% (GD), Severe Skewing (80–90%): 24.6% (NF) / 23.7% (GD), Extreme Severe Skewing (90–100%): 0.2% (NF) / 0.4% (GD). Severe skewing or higher (80–100%) was 24.8%/24.1% in NF/GD. Skewing or higher (70–100%) was 54.2%/51.1% in NF/GD. NF, normal female; GD, Grave’s disease

We calculated XCI skewing (see Materials and Methods for formula). Among 3,580/3,380 in NF/GD, 1,641/1,656 positions (45.8%/48.9%) showed relatively random sequencing (50–70%, R), 1,051/914 positions (29.4%/27.0%) showed skewing (70–80%, S), 880/803 positions (24.6%/23.7%) showed severe skewing (80–90%, SS), and 8/15 positions (0.2%/0.4%) showed extreme severe skewing (90–100%, E) (Fig. 2).

Compared with common SNPs of the NF and GD cells, 25 SNPs in 13 genes (dedicator of cytokinesis 11 (*DOCK11*, Gene ID: 139818), PABIR family member 3 (*FAM122C*, Gene ID: 159091), FTX transcript, XIST regulator (*FTX*, Gene ID: 100302692), G protein nucleolar 3 like (*GNL3L*, Gene ID: 54552), GTP binding protein 6 (putative) (*GTPBP6*, Gene ID: 8225), moesin (*MSN*, Gene ID: 4478), phosphorylase kinase regulatory subunit alpha 2 (*PHKA2*, Gene ID: 5256), protein kinase cAMP-dependent X-linked catalytic subunit (*PRKX*, Gene ID: 5613), retinitis pigmentosa GTPase regulator (*RPGR*, Gene ID: 6103), transducin beta like 1 X-linked (*TBL1X*, Gene ID: 6907), THO complex subunit 2 (*THOC2*, Gene ID: 57187), three prime repair exonuclease 2 (*TREX2*, Gene ID: 11219), tRNA methyltransferase 2 homolog B (*TRMT2B*, Gene ID: 79979), ubiquitin specific peptidase 9 X-linked (*USP9X*, Gene ID: 8239), and zinc finger protein X-linked (*ZFX*, Gene ID: 7543)) were significantly associated with each other by the Z-score test and the χ2 test. Twelve SNPs in GD had greater skewing than NF and thirteen had less skewing. When adopting the false discovery correction method, two SNPs in the *FAM122C* gene showed the highest significance (*p* < 0.001) (Table S3).

### Possible XCI escape genes

**Fig. 3:**
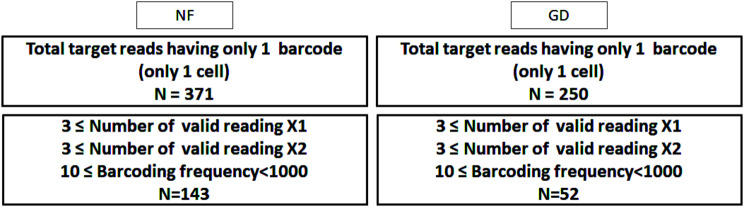
Strategy collected from total target reads having only 1 barcode to target positions of NF and GD for XCI escape. Of the total 371/250 nucleotide positions were filtered only one barcode (representing a unique cell). 143/52 were identified as heterozygous with a barcoding frequency from 10 to 1,000 times and more than three effective supporting reads from both alleles (X1 and X2). NF, normal female; Grave’s disease; X1, one allele of heterozygous genotype; X2, another allele of heterozygous genotype

We established common criteria for analyzing XCI escape. Of the total 371/250 nucleotide positions with only one barcode (representing a unique cell), 143/52 were identified as heterozygous, with a barcoding frequency from 10 to 1,000 times and more than three effective supporting reads from both alleles (X1 and X2) (Fig. 3). One allele of a heterozygous genotype (X1) was 143/52 of target positions, 3/3 of minimum, 16/15 of maximum, 5.79/7.92 of mean, and 2.91/3.53 of standard deviation. Another allele of the heterozygous genotype (X2) was 143/52 of target positions, 3/3 of minimum, 23/16 of maximum, 9.51/7.21 of mean, and 5.74/4.29 of standard deviation. The barcoding frequency was 143/52 of target positions, 10/11 of minimum, 40/27 of maximum, 17.41/15.69 of mean, and 6.79/4.87 of standard deviation. The barcode number was 143/52 of target positions, 1/1 of minimum, 1/1 of maximum, 1/1 of mean, and 0.00/0.00 of standard deviation (Table 3). These 143/52 nucleotide positions spanned 20/6 genes, comprising the 12 NF genes apolipoprotein O (*APOO*, Gene ID: 79135), calcium/calmodulin dependent serine protein kinase (*CASK*, Gene ID: 8573), cyclin dependent kinase like 5 (*CDKL5*, Gene ID: 6792), dehydrogenase/reductase X-linked (*DHRSX*, Gene ID: 207063), ectodysplasin A (*EDA*, Gene ID: 1896), *GTPBP6*, *MSN*, *PRKX*, SH3 domain containing kinase binding protein 1 (*SH3KBP1*, Gene ID: 30011), steroid sulfatase (*STS*, Gene ID: 412), zinc finger protein 81 (*ZNF81*, Gene ID: 347344), and zinc finger CCCH-type, RNA binding motif and serine/arginine rich 2 (*ZRSR2*, Gene ID: 8233) and the GD gene *EDA* (Table 4).

**Table 3:** Data interpretation for assessment of XCI escape

	NF/GD				
	Target positions	Minimum	Maximum	Mean	Standard deviation
X1	143/52	3/3	16/15	5.79/7.92	2.91/3.53
X2	143/52	3/3	23/16	9.51/7.21	5.74/4.29
Barcoding frequency	143/52	10/11	40/27	17.41/15.69	6.79/4.87
Number of barcode	143/52	1/1	1/1	1/1	0.00/0.00
Validation	143/52				

NF, normal female; GD, Grave’s disease; X1, one allele of heterozygous genotype; X2, another allele of heterozygous genotype

**Table 4:** XCI escape genes (alphabetical order)

NF		GD	
Gene name	Previous study [21, 65]	Gene name	Previous study [21, 65]
*AMMECR1*		*DIAPH2*	
*APOO*	+	*EDA*	+
*ARMCX5-GPRASP2*		*IL3RA*	
*CASK*	+	*MED14*	
*CDKL5*	+	*PPP2R3B*	
*DHRSX*	+	*TXLNG*	
*EDA*	+		
*GTPBP6*	+		
*LINC00630*			
*MSN*	+		
*PGK1*			
*PHF8*			
*PRKX*	+		
*SH3KBP1*	+		
*STAG2*			
*STS*	+		
*TAF1*			
*TXLNG*			
*ZNF81*	+		
*ZRSR2*	+		

## Discussion

In this study, we observed XCI skewing and escape in both a healthy donor NF and in a patient with GD (Table S3) using scRNA-seq and Linux-based processing (Tables 1 and 2). The GD patient in this study, diagnosed at 16, has experienced over five recurrences during more than five years of ATD therapy, classified as intractable GD [51, 52]. We aimed to estimate XCI skewing and the occurrence of XCI escape using scRNA-seq of NF and GD cells. We also attempted to read exonic SNPs, XCI skewing and X chromosome target genes that cause female-biased development of GD cells. We revealed the associations between the SNP of immune-related genes’ XCI skewing ratio in NF and GD cells.

We established cut-off criteria for XCI skewing and escape (Figs. 1 and 3) and used these criteria to analyze the data (Tables 2 and 4). The degree of XCI skewing was determined using the formula in Fig. 2. We observed significant associations when comparing the degree of XCI skewing in NF and GD (Table S3). XCI escape gene names are shown in Table 4.

Previous studies analyzing the degree of XCI skewing used different cutoffs of XCI distortion (65–90%) to define skewed XCI [18, 29–33]. We defined the degree of XCI skewing as relatively random (50–70%), skewing (70–80%), severe skewing (80–90%), and extreme severe skewing (90–100%). The proportion of healthy controls with XCI skewing was previously shown to be high in Asian (Japan and South Korea), Indian, and Caucasian (Tunisia and Turkey) study populations, which were similar to those found in this study (3,580/3,380 in the NF/GD) (Table 2) [17, 18, 53–56]. One group hypothesized that XCI skewing influences autoimmune propensity in women [57]. However, we found no difference in the ratio of XCI skewing between AITD patients and control subjects. We did not find any differences in the effects of skewing severity on the development of GD compared to NF.

In total, we found 15 genes significantly associated with XCI skewing ratios of NF and GD: *DOCK11*, *FAM122C*, *FTX*, *GNL3L*, *GTPBP6*, *MSN*, *PHKA2*, *PRKX*, *RPGR*, *TBL1X*, *THOC2*, *TREX2*, *TRMT2B*, *USP9X*, and *ZFX*. *GTPBP6*, *PHKA2*, *PRKX*, *RPGR*, and *USP9X* were reported to show XCI or XCI skewing. Of these, the *FAM122C* gene showed the most significant skewing (*p* < 0.001). There is an Xq26.3–q28 deletion in primary ovarian insufficiency (POI) patients. Among them, *FAM122C* is a gene in which the expression of the gene has changed more than five-fold. GFP-positive cells differentiated from primary ovarian insufficiency-induced pluripotent stem cell (POI-iPSC) exhibited reduced expression of *FAM122C* than cells of normal iPSC [58, 59]. The interplay of genes like *FAM122C* can anticipate their collective contribution to immune dysregulation in GD, related to the differentiation of primordial germ cells [59]. *FAM122C* studies confirming a direct association with the immune system or autoimmune disease are lacking, and further studies are needed to understand the relationship between genes and immune modulation abnormalities. *GTPBP6* was related to overall IQ of XCI pattern [60]. Sequence analysis, in one case, revealed the leukocytes in an asymmetric XCI pattern in the expression of the GD patient’s *PHKA2* genetic mutation (c.3614 C>T; p.P1205L). Reports of X-linked GSDIXa in female carriers exhibiting asymmetric XCI are rare. Asymmetric XCI plays a key role in the expression of X-linked feel disorder in female carriers [61]. Patients with 46,XX testicular disorder exhibit a translocation between *PRKX* and reverse protein kinase Y (*PRKY*) genes [62]. In the study cohort, female X-linked *RPGR* gene demonstrated clinical severity. XCI ratios are related to clinical severity and disease [63]. A case report of intellectual disability (ID) showed that neuroligin 4 X-linked (*NLGN4X*, Gene ID: 57502), histone deacetylase 8 (*HDAC8*, Gene ID: 55869), TATA-box binding protein associated factor 1 (*TAF1*, Gene ID: 6872), and *USP9X* genes were associated with XCI escape. Thus, it seems that XCI skewing is a very good way to characterize molecular mechanisms based on X-linked Variants Causing Intellectual Disabilities (XLIDs) in women [64].

In total, 371/250 nucleotide positions that have only one barcode (representing a unique cell) were identified for XCI escape. Of these, 143/52 nucleotide positions spanned 20/6 genes, 12/1 of which (*APOO*, *CASK*, *CDKL5*, *DHRSX*, *EDA*, *GTPBP6*, *MSN*, *PRKX*, *SH3KBP1*, *STS*, *ZNF81*, and *ZRSR2*/*EDA*) were identified as XCI escapes in previous studies [21, 65]. DNA methylation of the *CDKL5* promoter, a gene responsible for neonatal epilepsy in human neuron-like cells, was edited from an asexual X-chromosomal allele to create an artificial escape. Three guide RNAs were used to confirm that fusion of catalytic domains from TET1 to dCas9 targeting the *CDKL5* promoter caused significant reactivation of inactive alleles, which allowed *CDKL5* to escape X-chromosome inactivation. Escape of X-chromosome inactivation is likely to be diagnosed in people with X-related disorders [66]. The *DIAPH2*, *EDA*, interleukin 3 receptor subunit alpha (*IL3RA*, Gene ID: 3563), mediator complex subunit 14 (*MED14*, Gene ID: 9282), *PPP2R3B*, and *TXLNG* genes are known to be involved in immune response, cell signaling, and gene expression regulation [21, 65, 67–70]. Overexpression of these genes due to XCI escape may disrupt normal immune tolerance mechanisms, thereby promoting the activation of autoreactive immune cells and the production of autoantibodies against thyroid antigens. This provides a reasonable explanation for the possible involvement of these genes in the pathogenesis of GD, and supports the need for further studies to confirm this association. *TLR7*, which was not identified in the present study, is located on the X chromosome, and XCI escapes allow it to show higher expression in women compared to men and is associated with a higher incidence of autoimmune diseases in women [71–73]. GD is an autoimmune disorder characterized by hyperthyroidism due to autoantibodies targeting the thyrotropin receptor (TSHR) [74]. The disorder shows a bias toward a CD4 + helper T cell type 2 (Th2) immune response [75] and overproduction of TSHR autoantibodies [74]. Recent several studies seem to be a potential connection between GD and XCI escape genes in terms of both susceptibility and pathophysiology as follows. AMMECR nuclear protein 1 (*AMMECR1*, Gene ID: 9949) can cause dysregulation of immune-related metabolic pathways, making it more susceptible to autoimmunity [76]. *APOO* may be involved in the development of GD and thyroid autoimmunity by causing mitochondrial dysfunction and oxidative stress [77, 78]. Abnormal immune cell death is a hallmark of autoimmunity, and it is possible that the ARMCX5-GPRASP2 readthrough (*ARMCX5-GPRASP2*, Gene ID: 100528062) gene is GD-associated [79, 80]. Although *CASK* is primarily associated with neural function, calcium signal dysregulation may contribute to changes in the immune response of GD [81]. *CDKL5* can cause abnormal kinase activity, affecting the proliferation and differentiation of immune cells [66, 82]. *DHRSX* can cause redox imbalances in thyroid cells, exacerbating antigen presentation and autoimmunity [83]. Aberrant immune signaling through pathways involving *EDA* could contribute to Th2-skewed responses [84]. Dysregulated *IL3RA* expression can amplify Th2 responses, aligning with GD pathology [85, 86]. *ZNF81* may cause changes in transcriptional regulation, resulting in an excessive immune response [87, 88]. *MED14* involved in unregulated transcription may affect the activation of immune cells and the production of antibodies in GD [89]. Aberrant signaling of *PPP2R3B* gene could alter T cell activity, promoting autoantibody production [90]. The higher expression of escape genes in females may contribute to the sex bias observed in autoimmune diseases like GD. Genes such as *IL3RA* and *EDA* likely promote the Th2 cytokine environment, enhancing TSHR autoantibody production. Genes like *APOO* and *ARMCX5*-*GPRASP2* contribute to mitochondrial dysfunction and immune dysregulation, core aspects of GD pathophysiology. The connection between these XCI escape genes and GD lies in their contributions to immune dysregulation, oxidative stress, and transcriptional imbalances, all of which amplify the autoimmune processes in GD.

These findings may have broader implications for the treatment or understanding of autoimmune diseases, such as SS, SLE, RA, and MS, which often have significant gender imbalances in pathogenesis, progression, and treatment outcome [10–14] and are thought to result from a mix of genetic, hormonal, and environmental factors [7, 8].

This study has some limitations. First, we focused on approximately 5,000 heterozygous nucleotide positions of the 155 million base pairs on the X chromosome, as selected by the Samtools phase program, and the results are only based on a partial examination of the X chromosome. Second, we could not include additional NF and GD samples to ensure robust comparisons. The study is conducted with a single NF and a single GD patient. Third, disproportionate X inactivation may be observed by tissue and age [91]. The further study will consider an age-matched control group. Since we analyzed distorted XCI in PBMCs, this study reflected only X inactivation imbalance in PBMCs at a specific time point and not in thyroid infiltrating cells. While scRNA-Seq provides a novel approach to studying XCI at single-cell resolution, the integration of long-read sequencing technologies could further enhance the detection of structural variants, repeat expansions, and epigenetic modifications, as previously demonstrated [92]. In the future, it would be better to compare the degree of XCI distortion in thyroid infiltrating cells. Clinical and experimental studies of them are needed to contribute to the understanding of the disease.

## Conclusion

Our results suggest that approximately 24.8% of the target nucleotide positions in NF and 24.1% of those in GD exhibit severe skewing or higher. Skewed XCI is not associated with the onset of GD, but it is associated with the degree of XCI skewing and with XCI escapes and GD in a few immune genes. We analyzed 4,000 base pairs (0.00003%) showing X chromosome heterozygsity through Samtools phase program and confirmed that XCI skewing is not relatively random, at about 70% in NF/GD, respectively. In addition, we confirmed XCI escape-related genes. To our knowledge, this is the first study to show simultaneous association of GD with XCI skewing and escape based on scRNA-seq. These results could potentially provide a method for detecting XCI skewing and escape using scRNA-seq. Due to the variability in the degree of skewing, additional research is necessary to investigate the correlation between these base pair variations, the clinical implications of this, and the skewing degree.

## Electronic supplementary material

Below is the link to the electronic supplementary material.

Supplementary Material 1

## Data Availability

The datasets generated and/or analysed during the current study are available in the Harvard dataverse repository, 10.7910/DVN/1KH0UH.
