# Supplementary material for: Assessment of XCI skewing and demonstration of XCI escape region based on single-cell RNA sequencing: comparison between female Grave’s disease and control
Source: BMC Mol Cell Biol. 2025 Jan 31;26:8. doi: 10.1186/s12860-025-00533-z (PMC11786500; doi:10.1186/s12860-025-00533-z)
Supplement: Supplementary file 1 — Supplementary Material 1 [file 12860_2025_533_MOESM1_ESM.docx]

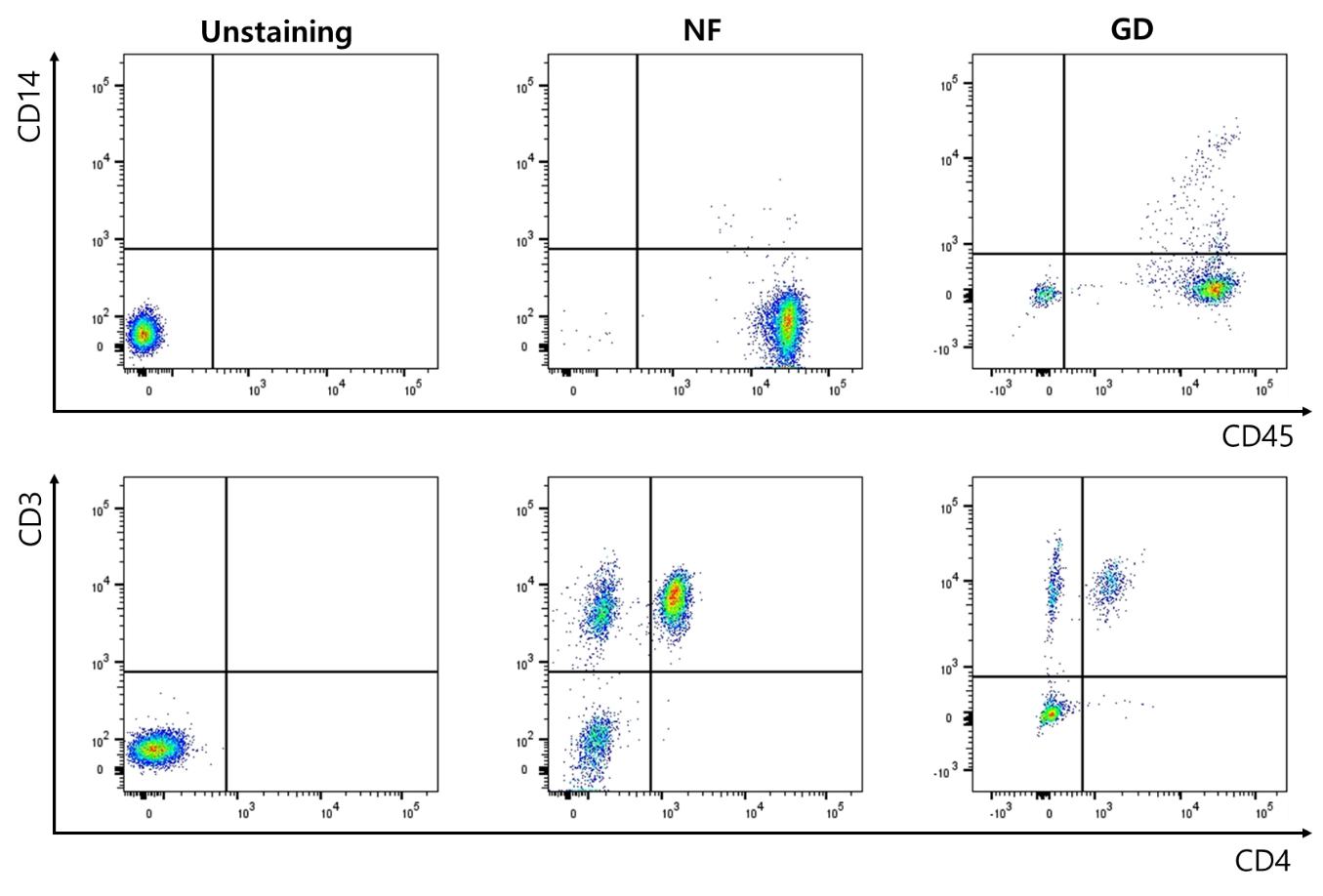
**Figure S1.** CD4+ T cells validation process. PBMCs were isolated from NF and GD. CD4+ T cells were confirmed by the flow cytometric analysis.

**Table S1.** Data processing codes in Ubuntu-based Linux PC from sequencing machinery to text files of NF and GD

| samtools view -b possorted_genome_bam.bam chrX > chrX_only.bam  samtools index chrX_only.bam  samtools phase -b phased chrX_only.bam  #phased output to txt file  samtools phase -b phased chrX_only.bam > phased_output.txt  samtools index phased.0.bam  samtools index phased.1.bam  samtools index phased.chimera.bam |
| --- |

**Table S2.** Data processing codes in Ubuntu-based Linux PC from text files to result files of NF and GD for the number of barcode and barcoding frequencies

| phaseFile="phased_output.txt"  bamFile="chrX_only.bam"  outputFile="result_HN00183217-gene.txt"  > "$outputFile"  while read -r line; do  if [[ $line == M1* ]] \|\| [[ $line == M2* ]]; then  read -r id chromosome startPos endPos allele0 allele1 hetIndex supports0 errors0 supp1 err1 <<<$(echo $line)  geneName=$(samtools view $bamFile $chromosome:$endPos-$endPos \| grep "GN:Z:" \| awk '{for(i=1;i<=NF;i++) if ($i ~ /^GN:Z:/) {split($i, a, ":"); print a[3]]}' \| sort \| uniq -c \| sort -nr \| head -n 1 \| awk '{print $2}')  start=$((endPos - 90))  end=$endPos  barcodeReads=$(samtools view $bamFile $chromosome:$start-$end \| awk -v start=$start -v end=$end '{pos=$4; if(pos >= start && pos <= end) print}')  barcodeReadCount=$(echo "$barcodeReads" \| wc -l)  uniqueBarcodes=$(echo "$barcodeReads" \| awk '{for(i=1;i<=NF;i++) if ($i ~ /^CB:Z:/) {split($i, a, ":"); print a[3]}}' \| sort \| uniq \| wc -l)  maxSupport=$(($supports0 > $supp1 ? $supports0 : $supp1))  totalSupports=$(($supports0 + $supp1))  alleleRatio=0  if [ "$totalSupports" -ne 0 ]; then  alleleRatio=$(echo "scale=4; $maxSupport / $totalSupports" \| bc)  fi  echo "$line $geneName $start-$end $supports0 $supp1 $alleleRatio $barcodeReadCount $uniqueBarcodes" >> "$outputFile"  fi  done < "$phaseFile" |
| --- |

**Table S3.** Multiple test of XCI skewing values in NF and GD.

|  |  | X1/X2 | |  | NF | | |  | GD | | |  |  |  | Correction's methods | | | |  | OR | |
| --- | --- | --- | --- | --- | --- | --- | --- | --- | --- | --- | --- | --- | --- | --- | --- | --- | --- | --- | --- | --- | --- |
| Gene name | SNP location | Allele 1 | Allele 2 |  | X1 | X2 | Skewing% |  | X1 | X2 | Skewing% | Z-Score | χ^2^ | p-value | Bonferroni | Hochberg | Hommel | False Discovery |  | χ^2^ | False Discovery |
| *GTPBP6* | 312165 | T | C |  | 8 | 1 | 88.9 (SS) |  | 65 | 57 | 53.3 (R) | 2.076 | 4.308 | 0.038 | NS | NS | NS | NS |  | 0.1 | NA |
| *PRKX* | 3659169 | T | C |  | 51 | 2 | 96.2 (E) |  | 11 | 4 | 73.3 (S) | 2.760 | 7.616 | 0.006 | NS | NS | NS | NS |  | 0.1 | NA |
| *TBL1X* | 9509749 | G | A |  | 10 | 52 | 83.9 (SS) |  | 67 | 40 | 62.6 (R) | 2.918 | 8.512 | 0.004 | NS | NS | NS | NS |  | 0.3 | NA |
| *TBL1X* | 9509754 | G | A |  | 10 | 50 | 83.3 (SS) |  | 58 | 37 | 61.1 (R) | 2.940 | 8.641 | 0.003 | NS | NS | NS | NS |  | 0.3 | NA |
| *PHKA2* | 18942625 | T | C |  | 59 | 13 | 81.9 (SS) |  | 25 | 16 | 61.0 (R) | 2.454 | 6.021 | 0.014 | NS | NS | NS | NS |  | 0.3 | NA |
| *ZFX* | 24185718 | G | A |  | 4 | 20 | 83.3 (SS) |  | 9 | 11 | 55.0 (R) | 2.051 | 4.207 | 0.040 | NS | NS | NS | NS |  | 0.2 | NA |
| *RPGR* | 38325046 | T | C |  | 10 | 10 | 50.0 (R) |  | 17 | 3 | 85.0 (SS)+ | 2.363 | 5.584 | 0.018 | NS | NS | NS | NS |  | 5.7 | NA |
| *USP9X* | 41099782 | G | A |  | 0 | 23 | 100.0 (E) |  | 8 | 5 | 61.5 (R) | 3.205 | 10.273 | 0.001 | NS | NS | NS | NS |  | NA | NA |
| *USP9X* | 41199520 | A | G |  | 19 | 12 | 61.3 (E) |  | 8 | 0 | 100.0 (E)+ | 2.115 | 4.473 | 0.034 | NS | NS | NS | NS |  | NA | NA |
| *USP9X* | 41230350 | T | G |  | 39 | 16 | 70.9 (S) |  | 0 | 13 | 100.0 (E)+ | 2.224 | 4.946 | 0.026 | NS | NS | NS | NS |  | NA | NA |
| *GNL3L* | 54563789 | G | A |  | 24 | 12 | 66.7 (R) |  | 29 | 4 | 87.9 (SS)+ | 2.086 | 4.350 | 0.037 | NS | NS | NS | NS |  | 3.6 | NA |
| *MSN* | 65702178 | G | A |  | 8 | 39 | 83.0 (SS) |  | 6 | 6 | 50.0 (R) | 2.397 | 5.745 | 0.017 | NS | NS | NS | NS |  | 0.2 | NA |
| *FTX* | 74276026 | T | C |  | 64 | 21 | 75.3 (S) |  | 33 | 3 | 91.7 (E)+ | 2.065 | 4.263 | 0.039 | NS | NS | NS | NS |  | 3.6 | NA |
| *TRMT2B* | 101047710 | T | C |  | 13 | 2 | 86.7 (SS) |  | 14 | 15 | 51.7 (R) | 2.284 | 5.216 | 0.022 | NS | NS | NS | NS |  | 0.2 | NA |
| *DOCK11* | 118679068 | G | A |  | 8 | 10 | 55.6 (R) |  | 2 | 18 | 90.0 (SS)+ | 2.408 | 5.797 | 0.016 | NS | NS | NS | NS |  | 7.2 | NA |
| *THOC2* | 123711650 | T | G |  | 9 | 0 | 100.0 (E) |  | 8 | 6 | 57.1 (R) | 2.284 | 5.219 | 0.022 | NS | NS | NS | NS |  | NA | NA |
| *FAM122C* | 134832611 | G | A |  | 6 | 107 | 94.7 (E) |  | 35 | 30 | 53.8 (R) | 6.532 | 42.666 | **< 0.001** | **< 0.001** | **< 0.001** | **< 0.001** | **< 0.001** |  | 0.1 | 0.1 |
| *FAM122C* | 134844028 | A | G |  | 13 | 15 | 53.6 (R) |  | 4 | 18 | 81.8 (SS)+ | 2.093 | 4.381 | 0.036 | NS | NS | NS | NS |  | 3.9 | NA |
| *FAM122C* | 134844029 | G | A |  | 8 | 24 | 75.0 (S) |  | 0 | 32 | 100.0 (E)+ | 3.024 | 9.143 | 0.003 | NS | NS | NS | NS |  | NA | NA |
| *FAM122C* | 134844042 | A | G |  | 19 | 11 | 63.3 (R) |  | 8 | 44 | 84.6 (SS)+ | 2.200 | 4.840 | 0.028 | NS | NS | NS | NS |  | 3.2 | NA |
| *FAM122C* | 134844054 | A | G |  | 14 | 15 | 51.7 (R) |  | 8 | 36 | 81.8 (SS)+ | 2.742 | 7.519 | 0.006 | NS | NS | NS | NS |  | 4.2 | NA |
| *FAM122C* | 134844064 | A | G |  | 25 | 36 | 59.0 (R) |  | 9 | 32 | 78.0 (S)+ | 1.999 | 3.997 | 0.046 | NS | NS | NS | NS |  | 2.5 | NA |
| *FAM122C* | 134844113 | A | G |  | 48 | 33 | 59.3 (R) |  | 11 | 86 | 88.7 (SS)+ | 4.528 | 20.504 | **< 0.001** | **0.001** | **0.001** | **0.001** | **< 0.001** |  | 5.4 | 5.4 |
| *TREX2;HAUS7* | 153459126 | C | T |  | 6 | 41 | 87.2 (SS) |  | 10 | 6 | 62.5 (R) | 2.176 | 4.736 | 0.030 | NS | NS | NS | NS |  | 0.2 | NA |
| *TREX2;HAUS7* | 153459158 | C | T |  | 0 | 22 | 100.0 (E) |  | 10 | 6 | 62.5 (R) | 3.130 | 9.797 | 0.002 | NS | NS | NS | NS |  | NA | NA |

X1, one allele of heterozygous genotype

X2, another allele of heterozygous genotype

R, the degree of XCI skewing, relatively random (50-70%)

S, the degree of XCI skewing, skewing (70-80%)

SS, the degree of XCI skewing, severe skewing (80-90%)

E, the degree of XCI skewing, extreme severe skewing (90-100%)

+, more degree of skewing than NF

NS, not significant

NA, not applicable
